# Swelling and Shrinking Properties of Thermo-Responsive Polymeric Ionic Liquid Hydrogels with Embedded Linear pNIPAAM

**DOI:** 10.3390/ijms15045337

**Published:** 2014-03-27

**Authors:** Simon Gallagher, Larisa Florea, Kevin J. Fraser, Dermot Diamond

**Affiliations:** 1 CLARITY, the Centre for Sensor Web Technologies, National Centre for Sensor Research, School of Chemical Sciences, Dublin City University, Dublin 9, Ireland; E-Mail: simon.gallagher24@mail.dcu.ie; 2 Insight Centre for Data Analytics, Dublin City University, Dublin 9, Ireland; E-Mails: larisa.florea@dcu.ie (L.F.); kevin.fraser@dcu.ie (K.J.F.)

**Keywords:** polymeric ionic liquids, ionic liquid, pNIPAAM, thermo-response, swelling, IPNs

## Abstract

In this study, varying concentrations of linear pNIPAAM have been incorporated for the first time into a thermo-responsive polymeric ionic liquid (PIL) hydrogel, namely tributyl-hexyl phosphonium 3-sulfopropylacrylate (P-SPA), to produce semi-interpenetrating polymer networks. The thermal properties of the resulting hydrogels have been investigated along with their thermo-induced shrinking and reswelling capabilities. The semi-interpenetrating networks (IPN) hydrogels were found to have improved shrinking and reswelling properties compared with their PIL counterpart. At elevated temperatures (50–80 °C), it was found that the semi-IPN with the highest concentration of hydrophobic pNIPAAM exhibited the highest shrinking percentage of ~40% compared to the conventional P-SPA, (27%). This trend was also found to occur for the reswelling measurements, with semi-IPN hydrogels producing the highest reswelling percentage of ~67%, with respect to its contracted state. This was attributed to an increase in water affinity due to the presence of hydrophilic pNIPAAM. Moreover, the presence of linear pNIPAAM in the polymer matrix leads to improved shrinking and reswelling response compared to the equivalent PIL.

## 1. Introduction

Ionic Liquids are classified as organic salts that possess melting points below 100 °C [1]. They have gained considerable attention in recent years due to their distinctive properties, including very low vapour pressure, good thermal stability and high ion conductivity [2–4]. An innovation that has arisen in recent years is a subclass of ILs called polymeric ionic liquids (PILs), prepared via polymerization of ionic liquid monomers, to form a solid, macromolecular structure.

The main features of PILs are that they combine all the unique properties associated with ILs, while being able to form coatings, gels, films and membranes due to their polymeric nature. Many different kinds of PILs have been designed and have found applications as functional polymers, namely solid polymer electrolytes [5], lithium batteries [6], solar cells [7], biomedical [8] and electrochromic devices [9].

Recently, a new class of PILs displaying thermo-responsive behaviour has been reported [10–13]. The development of these PILs creates possibilities for new applications such as selective extraction of water-soluble proteins [10,12,14]. The first reported thermo-responsive PIL to display phase separation at elevated temperatures *i.e.*, LCST (Lower Critical Solution Temperature) behaviour, consisted of a tetrabutyl-phosphonium cation and 4-styrene sulfonate anion [15]. When dissolved in water, this PIL precipitated at a temperature higher than its LCST, and re-dissolved when cooled below the LCST, much like the thermo-responsive polymer *N-*isopropylacrylamide (pNIPAAM) [16]. Following this report, several thermo-responsive PILs have been developed based on variations of phosphonium cations, such as tetrabutyl or tributyl-hexyl, combined with anion derivatives based on benzenesulfonic acid [10,12,13,17].

Recently, we reported the polymerization of thermo-responsive monomeric ILs and subsequent formation of thermo-responsive PIL hydrogels [18]. In this work, we established suitable cross-linkers for the production of mechanically robust PIL hydrogels and investigated their endothermic transition behaviour. The cross-linked PILs were found to display a significant broadening of the temperature range over which LCST behaviour occurs compared with conventional thermo-responsive polymers like pNIPAAM.

However, the use of these temperature sensitive PIL hydrogels for applications such as polymer-based microfluidic valves is limited, because of their relatively slow response rate to temperature changes. Previous studies have proposed ways to improve the actuation kinetics of pNIPAAM-based hydrogels [19–25]. One of the most effective strategies has been the use of semi-interpenetrating networks (semi-IPNs) [20,24–26]. Semi-IPNs are composed of two or more chemically distinct networks wherein one of the polymer components is cross-linked while the other is in its linear form. For example, linear pNIPAAM has been integrated into cross-linked hydrogel networks, to examine whether its rapid response rate could improve the shrinking and reswelling capabilities of the cross-linked polymer [16,20].

**Figure 1.**
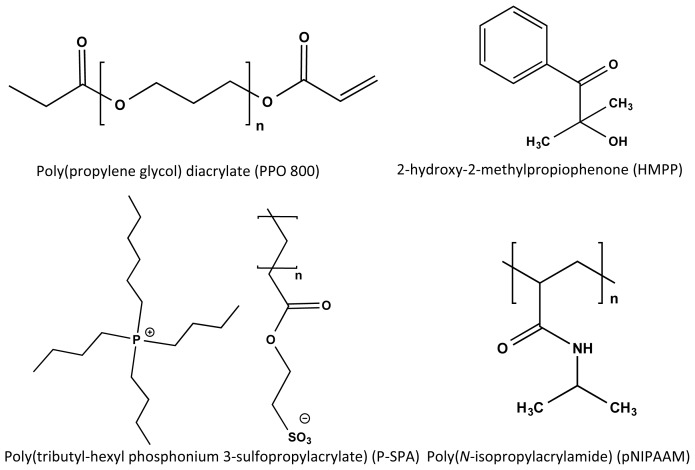
Components used in the formulation of semi-interpenetrating networks (IPN) hydrogels used in this study.

In this study, a cross-linked tributyl-hexyl phosphonium 3-sulfopropylacrylate (P-SPA) (Figure 1) hydrogel has been prepared with controlled amounts of linear pNIPAAM embedded in the hydrogel network in order to improve its actuation response characteristics. The resulting semi-IPN structures were investigated by measuring the shrinking behaviour of the hydrogels as a function of temperature and the reswelling kinetics at room temperature. DSC (differential scanning calorimeter) was used to further understand the influence of pNIPAAM concentration on the PIL LCST behaviour.

## 2. Results and Discussion

### 2.1. Morphological Properties of Hydrogels

**Figure 2.**
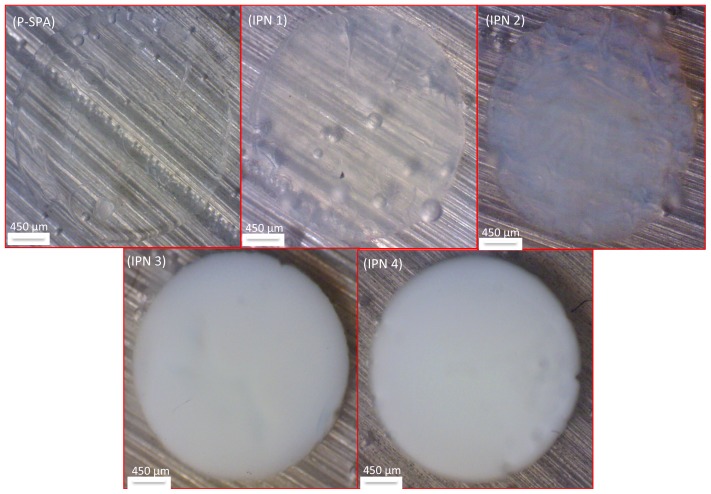
Hydrogels after polymerization in a circular mould (1 mm deep, 3 mm diameter) and subsequent swelling in deionized water at room temperature for 72 h, followed by contraction in deionized water at 40 °C for approximately 2 min.

The feed compositions and sample ID of the semi-IPNs are summarized in Table 1. During the crosslinking process, the monomeric IL and linear pNIPAAM chains form a semi-IPN network. To our knowledge, this is the first time linear pNIPAAM has been embedded in a thermo-responsive PIL hydrogel. In such a network, molecular interactions, such as hydrogen bonding, have been previously shown to occur between distinct polymers [27,28]. Such interactions can result in different morphological, thermal and actuation properties manifested by the semi-IPN compared to traditional pNIPAAM polymers. Figure 2 shows examples of hydrogels with composition ranging from 1:0 to 1:1.2 P-SPA:pNIPAAM mol % ratio after removal from a 3 mm in diameter polydimethylsiloxane (PDMS) mould and exposure to water. In preliminary results, hydrogels exceeding 1:1.2 P-SPA:pNIPAAM mol % ratio were found to fragment when swollen in water. Therefore investigations into LCST swelling/contraction behavior were restricted to hydrogels of composition 1:0 to 1:1.2 P-SPA:pNIPAAM mol % ratio, as this represented the range over which suitable physical robustness could be formed.

The images were recorded at 40 °C. Under these conditions, the pNIPAAM becomes hydrophobic and forms clathrate structures within the gels, which in turn produces an opaque appearance, as water is no longer bound to pNIPAAM [16]. Clearly, the opaque appearance of the gel increases as the mol % pNIPAAM is increased.

The hydrogels polymerized in a PDMS mould 1 mm deep and 3 mm in diameter appeared to be mechanically stable in their swollen state after hydration. For example, they did not fragment during the experiments and handling was relatively simple during shrinking and reswelling measurements (Figure 2).

### 2.2. Thermal Behaviour of Hydrogels

**Figure 3.**
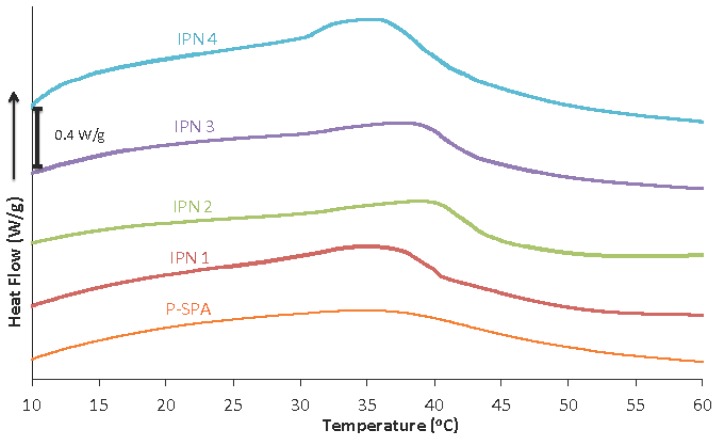
DSC endothermic transitions of hydrogels at heating rate 10 °C/min.

In our previous study, we found that when cross-linked, the thermo-responsive P-SPA exhibited a broad endothermic transition in contrast to the more narrow peak of its linear form [18]. This phenomenon was attributed to the decreased level of freedom due to the bulky and highly charged nature of the PIL when in the cross-linked hydrogel state. Figure 3 illustrates that all of the hydrogels analysed in this study exhibit broad LCST transitions, in contrast to linear pNIPAAM, which has a relatively sharp LCST typically appearing around 30–35 °C [16]. The DSC profile for P-SPA is broad and featureless, with a maximum in the range 34–37 °C. In contrast, the hydrogels that containing linear pNIPAAM show a distinct feature in the range 34–40 °C superimposed on the broad P-SPA profile, which is due to the presence of the pNIPAAM (Table S1). This suggests that the PIL and pNIPAAM components are, to some extent, retaining their independent characteristics within the semi-IPN hydrogels.

### 2.3. Shrinking Behaviour of Hydrogels

Prior to the shrinking experiments the hydrogels were allowed to swell in DI water as described in the experimental section. Although the initial diameter after polymerization for all of the hydrogels was 3 mm (equal to the diameter of the mould), they were found to exhibit different degrees of swelling (Table 2). The swollen diameter increased with increasing pNIPAAM content with the following trend: P-SPA < IPN 1 < IPN 2 < IPN 3. For hydrogel IPN 4, a reduction in the degree of swelling was observed relative to IPN 3, suggesting that there is an optimum amount of pNIPAAM in the hydrogel matrix around that of IPN 3 that maximises the water uptake. This effect may be due to the increasing concentration of pNIPAAM polymer chains decreasing the elasticity of the hydrogels, which in turn suppress the water uptake [25,29].

Table 2 shows the diameter of the contracted hydrogels at 75 °C and their subsequent shrinking percentage with respect to their fully swollen diameter prior to shrinking. The trend in shrinking percentage follows the trend, P-SPA < IPN 1 < IPN 2 < IPN 3 < IPN 4. The hydrophobic nature of pNIPAAM, above its normal LCST of ~35 °C [16], seems to have an effect on the semi-IPNs, increasing the degree of contraction. Above the LCST, hydrophobic interactions between isopropyl groups of pNIPAAM increase and polymeric chains start to aggregate and phase separation takes place. Entrapped water molecules are then freed due to broken hydrogen bonds, and the polymer collapses [16]. Previous studies have shown that the addition of linear pNIPAAM to a semi-IPN hydrogel increases shrinking at elevated temperatures [25]. The hydrogel IPN 4 is shown to have the greatest shrinking of 40.3% at 75 °C. This is a substantial increase compared to the 27.3% shrinking of P-SPA under the same conditions. This shows that a greater concentration of hydrophobic pNIPAAM in the hydrogel increases the shrinking percentage at elevated temperatures.

**Figure 4.**
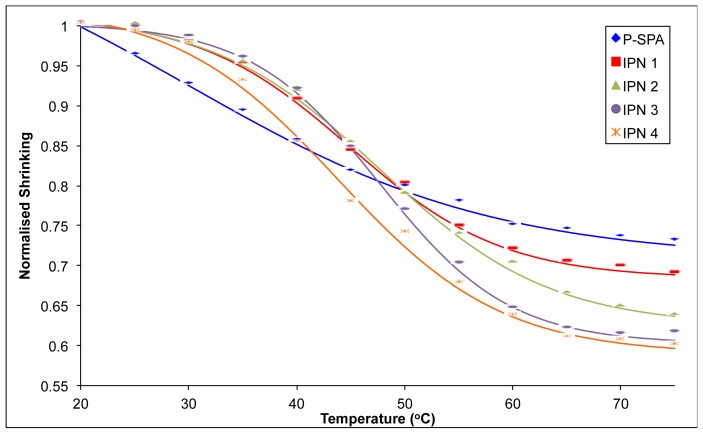
Temperature induced shrinking profiles of hydrogels from 20 to 75 °C at 5 °C increments, showing the best-fit of the experimental data (points) to a Boltzmann sigmoidal function (Equation (S1)) [30]. Shrinking monitored by the reduction of diameter size relative to the initial swollen diameter (Table 1).

Figure 4 shows the shrinking profiles of the hydrogels from their initial swollen state at 20 °C to their contracted state at 75 °C at 5 °C increments. The normalised shrinking values represent the percentage change of the contracted diameters compared to their initial swollen diameter value. The data shows that all the samples shrink as the temperature is increased from 20 to 75 °C, which is in agreement with the endothermic transitions in the DSC study. It can be seen that the shrinking profiles of the semi-IPNs are slightly steeper than that obtained for P-SPA. The slopes of the hydrogel shrinking-profiles (Figure 4) give a numerical indication of the extent of the shrinking effect as a function of temperature. The slopes were derived from the best fit of the experimental data sets to the Boltzmann sigmoidal function using Microsoft Excel Solver [30] and were found to follow the trend, P-SPA < IPN 1 < IPN 2 < IPN 4 < IPN 3 (Table 2). The data shows that the PIL-pNIPAAM IPN shrinks to a greater extent from 20 to 75 °C compared to the P-SPA. Previous studies have shown that the presence of pNIPAAM increases the number of pores in the morphology of semi-IPN hydrogels [20]. This leads to an increase in the surface area to bulk ratio, which in turn reduces the average diffusion pathlength for water within the material, and leads to more efficient movement of water into/out of the hydrogel. Therefore, the presence of pNIPAAM in the semi-IPNs produces a greater shrinking effect over this temperature range compared with P-SPA. Compared to the IPN samples, the P-SPA shrinking profile is very broad, and the effect very gradual over the entire temperature range studied, reaching *ca.* 27% reduction from the initial swollen diameter. In contrast, with all the IPN samples, the effect is much more pronounced in terms of the extent of shrinking, and the temperature range over which it occurs, with a steady state reached at *ca*. 60 °C. For example, IPN 3 shrinks by *ca*. 40% over the temperature range 30–60 °C.

From these data, it is apparent that the degree of temperature dependent contraction of the thermo-responsive PIL hydrogel can be controlled to some extent by varying the composition ratio of PIL/pNIPAAM in the semi-IPN material.

### 2.4. Reswelling Behaviour of Hydrogels

Table 3 shows the reswelling data of the hydrogels from their contracted state when placed in deionized water at 20 °C as a function of the diameter of the gel disc sample. The reswollen diameters are similar to their initial swollen diameters (Table 2) (±0.8%). This shows the gel reswelling behaviour is reasonably consistent with no observable hysteresis effect. The degree of reswelling follows the trend; P-SPA < IPN 1 < IPN 2 < IPN 3 < IPN 4; *i.e.*, the semi-IPNs display greater swelling capabilities than the standard P-SPA hydrogel. The hydrogel IPN 4 exhibited the highest swelling at 67.6% increase in diameter relative to its contracted state, which is nearly twice that of the P-SPA hydrogel, at 37.6% diameter increase. The increase in swelling is due to the increasing influence of linear pNIPAAM on the water uptake behaviour of the semi-IPN. The carbonyl and amide groups of pNIPAAM increase the hydrophilicity of the gel leading to an increase in water uptake and therefore greater tendency to swell in water.

**Figure 5.**
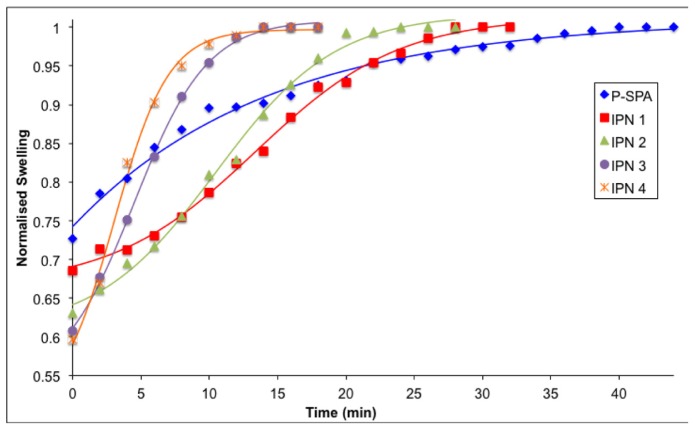
Reswelling profiles of hydrogels from contracted state at 20 °C as a function of time. Solid lines are best-fits to a Boltzmann sigmoidal function (Equation (S1)) [30].

In Figure 5, the reswelling profiles of the contracted hydrogels are shown as a function of time. The obtained diameter values were normalised by dividing them by the final reswollen diameter value. The contracted gels were placed in water at 20 °C and measurements taken over a period of up to 45 min. It can be seen that the semi-IPNs cease reswelling much earlier than the standard P-SPA. The slope of the reswelling profiles follows the trend; P-SPA < IPN 1 < IPN 2 < IPN 3 < IPN 4 (Table 3). The hydrogel P-SPA reaches its fully hydrated state after ~40 min, and the profile is very broad and gradual. In contrast, with IPN 3 and 4 the processes are essentially complete after ~14 min, and the extent of the swelling is greater at *ca.* 40% in both cases compared to *ca.* 25% for P-SPA after 35 min. This behaviour is consistent with the shrinking behaviour discussed earlier, which is ascribed to the increasing hydrophilicity and porosity as the % pNIPAAM increases in the semi-IPN.

## 3. Experimental Section

### 3.1. Chemicals and Materials

*N*-isopropylacrylamide 98% (NIPAAM), 2-hydroxy-2-methylpropiophenone 97% (HMPP), Potassium 3-sulfopropylacrylate (SPA), and poly(propylene glycol) diacrylate Mn 800 (PPO 800) were purchased from Sigma Aldrich^®^ (Wicklow, Ireland), and used as received. Tetrabutylphosphonium chloride ([P_4,4,4,4_][Cl]) (Cyphos 443W) and tributyl-hexyl phosphonium chloride ([P_4,4,4,6_][Cl]) were supplied by Cytec^®^ (Niagara Falls, ON, Canada) which were column cleansed using aluminium (activated, basic, Brockmann I) with dichloromethane used as the mobile phase. This was then removed under vacuum at 40 °C for 48 h at 0.1 Torr [31].

### 3.2. Synthesis of pNIPAAM

The required amount of NIPAAM and HMPP (2 mol %) were dissolved in a vial containing 1:1 volume ratio of distilled water and ethanol. The solution was then stirred until homogenisation. The vial was then placed in a UV curing chamber (λ = 365 nm, 3.5 mW/cm^2^) for 20 min. The resulting pNIPAAM was then purified by precipitation in water at 60 °C.

### 3.3. Synthesis of Tributyl-Hexyl Phosphonium 3-Sulfopropylacrylate (P-SPA)

The IL monomer, namely Tributyl-hexyl phosphonium 3-sulfopropylacrylate (P-SPA) was synthesized according to previous studies [18]. Seven grams of phosphonium chloride was mixed with 10 g of water and 1.2 molar equivalents of the anion salt ([Na][4-styrenesulfonate] for [P_4,4,4,4_][Cl] and [K][3-sulfopropylacrylate] for [P_4,4,4,6_][Cl], respectively). The mixture was stirred at room temperature for 48 h. The IL was extracted from the water phase by dichloromethane (DCM). The DCM phase was reduced by a rotary evaporator and the residual liquid was dried at high vacuum (0.1 mBar) for 24 h at room temperature. A final product yield of 95% was obtained.

**P-SPA** ^1^H NMR, δ_H_ (400 MHz, CDCl_3_): 0.75–0.79 (t, 3H, CH_3_), 0.84–0.88 (t, 9H, CH_3_), 1.19–1.22 (m, 4H, CH_2_), 1.38–1.44 (m, 16H, CH_2_), 2.07–214 (m, 2H, CH_2_), 2.15–2.24 (m, 8H, CH_2_), 2.75–2.79 (m, 2H, CH_2_), 4.13–17 (t, 2H, CH_2_), 5.68–5.71 (dd, 1H, CH), 5.93–6.00 (q, 1H, CH), 6.23–6.28 (dd, 1H, CH) ppm.

### 3.4. Preparation of Semi-IPN Hydrogel

The synthesized pNIPAAM was added to a vial containing the required amount of P-SPA, PPO 800 (5 mol %) and HMPP (2 mol %). For each gel, the mole ratios for NIPAAM, crosslinker and initiator were calculated with regard to the IL monomer content. The contents of the vial were stirred with a magnetic stirrer until the pNIPAAM was dissolved and the result solution was homogenised. The solution was then quickly cast into a PDMS mould. The gels were polymerised for 40 min in a UV curing chamber that produced 365 nm UV light intensity of 3.5 mW/cm^2^.

These semi-IPN structures were then immersed in deionized water at room temperature for at least 72 h to reach equilibrium and to extract any unreacted monomers. The water was refreshed every several hours during this treatment. The feed compositions and sample ID of the reaction are summarized in Table 1.

### 3.5. Thermobehaviour of Semi-IPN Hydrogels

The LCST behaviour of the swollen gels was monitored on a Q200 DSC TA instrument. Thermal scans below room temperature were calibrated with the cyclohexane solid-solid transition and melting point at −87.0 and 6.5 °C, respectively. Thermal scans above room temperature were calibrated using indium, tin and zinc with melting points at 156.6, 231.93 and 419.53 °C, respectively. Approximately 10 mg of the water-swollen samples were placed on a tissue to remove excess water and were weighed on a microscale before the DSC measurement. These samples were then placed on aluminium DSC plates and sealed. The LCST values for these samples were determined by thermal scans at 10 °C/min with the following temperature program: Heat from 0 to 90 °C then cool from 90 to 0 °C. This process was repeated three times in order to observe if the endothermic process was reproducible. The LCSTs were determined as the endothermic peak during heating.

### 3.6. Measurement of Shrinking of Semi-IPN Hydrogels

After polymerisation in the PDMS mould (1 mm deep and 3 mm diameter), the gels were allowed to fully swell for 72 h in deionized water at 20 °C, before the shrinking measurements were taken. The imaging was performed using an Aigo GE-5 microscope with a 60× objective lens and the accompanying Aigo ScopeImage 9.0 software (Aigo, Beijing, China). The gels were placed on an aluminium plate resting on an Anton Paar MCR 301 Rheometer peltier holder (Anton Paar, Graz, Austria). The plate was filled with DI water and covered with a glass plate to avoid evaporation of water. The glass plate was also in contact with the filling water to avoid condensation. Temperature of the holder was controlled by the rheometer software and was ramped up from 20 to 75 °C by 5 °C steps. During each step the temperature around the sample was checked with a Fluke 62 Mini IR thermometer (Everett, WA, USA) and the diameter of the gel was taken once the temperature stabilised after approximately 2 min. This experiment was performed for three samples of each type of gel. The microscope software was used to measure the diameter of the gel and this value was compared to a diameter value obtained using a MATLAB R2010a program (The MathWorks, Natick, MA, USA) for validation purposes (Figure S1). For better visualisation, the experimental data was fitted using a Boltzmann sigmoidal function (Equation (S1)) using Microsoft EXCEL Solver [30]. An example can be found in the supplementary information (Figure S2).

### 3.7. Measurement of Reswelling of Semi-IPN Hydrogels

The contracted gels were placed in deionized water on the Anton Paar MCR 301 Rheometer peltier holder. Temperature was controlled by the holder rheometer software and was kept at 20 °C throughout the experiment. Images were taken using an Aigo GE-5 microscope with a 60× objective lens and the accompanying software (Beijing, China). Images were taken every two minutes until the gel swelling appeared to stop. This experiment was performed for three samples of each type of gel. The diameter of the gel was measured as described in Section 3.6. For better visualisation, the experimental data was fitted using a Boltzmann sigmoidal function (Equation (S1)) using Microsoft EXCEL Solver [30].

## 4. Conclusions

In this study, cross-linked thermo-responsive PIL hydrogels were prepared with different concentrations of linear pNIPAAM chains incorporated in each gel, producing novel semi-IPN networks. The DSC data showed that the presence of pNIPAAM caused a narrowing of the broad LCST transition shown by the PIL hydrogel, which was attributed to the fast response nature of the linear pNIPAAM chains.

At elevated temperatures, the presence of hydrophobic pNIPAAM within the semi-IPN network provided an increased shrinking percentage of 40.3% compared to the respective 27.3% of P-SPA. Moreover, the hydrophilic presence of pNIPAAM provided a platform for higher and quicker reswelling at room temperature. These novel semi-IPN hydrogels are found to exhibit interesting swelling/contraction trends with respect to linear pNIPAAM concentration within the gel. In terms of producing gels with the best actuation characteristics for potential applications such as flow control in microfluidics, a composition around that of IPN 3/IPN 4 would appear to be optimum, as these gels show the largest swollen diameters, the largest degree of shrinking, and the most rapid rate of reswelling (compare data in Tables 2 and 3).

## Supplementary Information



## Figures and Tables

**Table 1.** Compositions of the semi-IPN Hydrogels.

Materials	Sample ID				

	P-SPA	IPN 1	IPN 2	IPN 3	IPN 4
P-SPA (μmol)	800	800	800	800	800
pNIPAAM (μmol)	0	240	480	800	960
PPO 800 a (μmol)	40	40	40	40	40
HMPP b (μmol)	16	16	16	16	16
P-SPA:pNIPAAM (mol ratio)	1:0	1:0.3	1:0.6	1:1	1:1.2

a 5 mol % concentration with respect to PIL;

b 2 mol % concentration with respect to the PIL.

**Table 2.** Shrinking properties of hydrogels at 75 °C.

Sample	Initial Swollen Diameter a (mm)	Contracted Gel Diameter b (mm)	% Shrinking c	Slope d
P-SPA	5.33 (0.07)	3.88 (0.07)	27.2	−0.007
IPN 1	5.61 (0.01)	3.85 (0.06)	31.2	−0.011
IPN 2	6.11 (0.17)	3.88 (0.11)	36.4	−0.012
IPN 3	6.22 (0.07)	3.81 (0.14)	38.7	−0.016
IPN 4	5.73 (0.09)	3.43 (0.04)	40.2	−0.014

a Initial swollen diameter of gel after 72 h in deionized water when temperature is 20 °C (*n* = 3);

b Contracted diameter of gel from fully hydrated state when temperature is 75 °C (*n* = 3);

c Percentage of shrinking of gel with respect to initial swollen diameter;

d The slope of the reduction in diameter (mm) *vs.* temperature step (5 °C) for the data presented in Figure 4 calculated using the Boltzmann sigmoidal function (Equation (S1)) [30].

Standard deviations are given in parentheses.

**Table 3.** Reswelling properties of hydrogels at room temperature.

Sample	Contracted Gel Diameter a (mm)	Reswollen Gel Diameter b (mm)	% Reswelling c	Slope d (min^−1^)
P-SPA	3.88 (0.07)	5.34 (0.08)	37.6	0.014
IPN 1	3.85 (0.06)	5.62 (0.01)	45.8	0.016
IPN 2	3.88 (0.11)	6.16 (0.16)	58.8	0.023
IPN 3	3.81 (0.14)	6.27 (0.06)	64.4	0.044
IPN 4	3.43 (0.04)	5.75 (0.09)	67.6	0.062

a Contracted diameter of gel at 75 °C (*n* = 3);

b Reswollen gel diameter from contracted state when temperature is 20 °C (*n* = 3);

c Percentage of reswelling of gel with respect to contracted gel diameter;

d The slope of the reswelling kinetics was calculated using the Boltzmann sigmoidal function (Equation (S1)) from the data in Figure 5 [30].

Standard deviations are given in parentheses.
