# Supplementary material for: Swelling and Shrinking Properties of Thermo-Responsive Polymeric Ionic Liquid Hydrogels with Embedded Linear pNIPAAM
Source: Int J Mol Sci. 2014 Mar 27;15(4):5337–49. doi: 10.3390/ijms15045337 (PMC4013567; doi:10.3390/ijms15045337)
Supplement: Supplementary file 1 [file ijms-15-05337-s001.pdf]

Supplementary Information

1. Example of Shrinking Measurement

Figure S1. Three examples of IPN 4 in deionized water at 40 °C captured with Aigo GE-5 microscope with a 60× objective lens and the diameter measured using Aigo ScopeImage 9.0 software. The measured diameters are then compared with a MATLAB R2010a program for validation purposes.

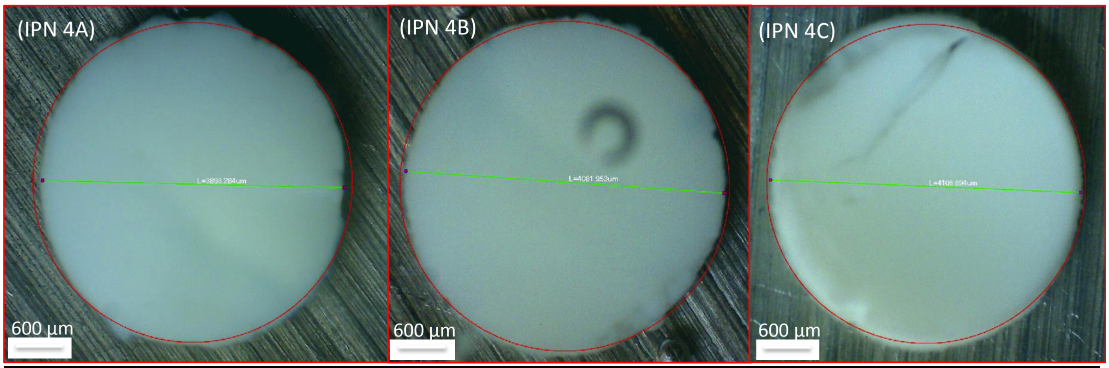

Sample	Microscope Diameter (mm)	MATLAB Circle Diameter (mm)
IPN 4A	3.898	4.123
IPN 4B	4.081	4.211
IPN 4C	4.106	4.258

2. Swelling and Reswelling Kinetics

The shrinking and reswelling kinetics were modeled using the Boltzmann sigmoidal function given below.

$$y = \frac{A_1 - A_2}{1 + e^{(x-x_0)/dx}} + A_2 \quad \text{(S1)}$$

where A_1 is the low Y limit, A_2 is the high Y limit, x_0 is the inflection (half amplitude) point and dx is the width.

For IPN1-4, the slope at x_0 was calculated with the formula: slope = $(A_2 - A_1)/4dx$, according to the BOLTZMANN equation.

For P-SPA the slope was calculated graphically as the data does not show an inflection point.

Example of fitting of experimental data of shrinking kinetics using Boltzmann sigmoidal function;

3. IPN 3

Adj. R-Square 0.9993

		Value	Standard Error
B	A1	1.00315	0.00333
B	A2	0.60235	0.00338
B	x0	47.67935	0.22474
B	dx	6.008	0.21836
Slope calculated = −0.0166			

Figure S2. Shrinking data of IPN 3 fitted using Boltzmann sigmoidal function.

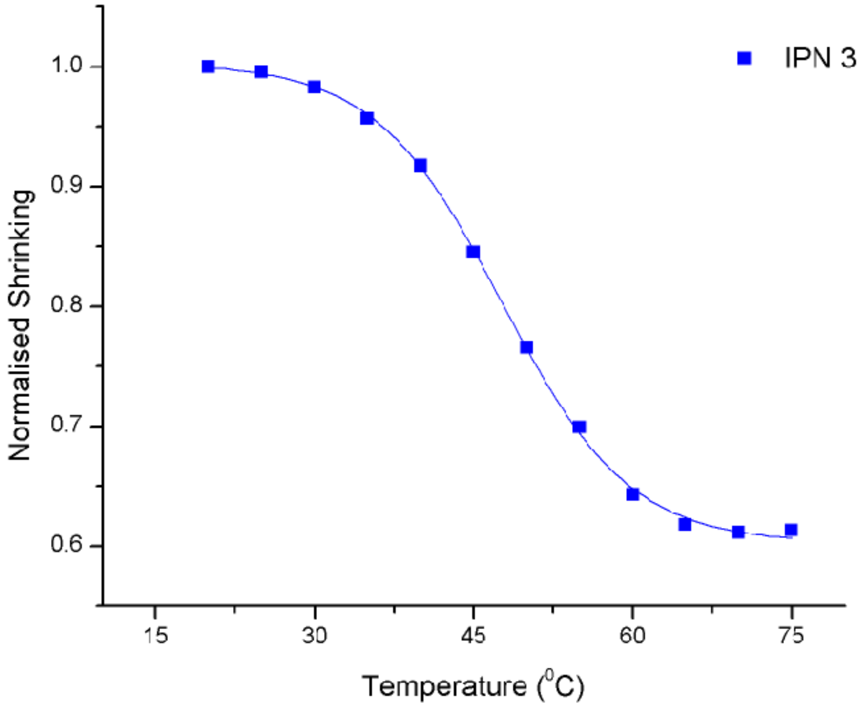

4. Thermal Behavior of Hydrogels

Table S1. LCST peak value ranges of hydrogels and subsequent comments.

Hydrogel	LCST (°C)	Comment
P-SPA	32.36–36.13	broad, featureless
IPN-1	33.49–35.87	Distinct feature
IPN-2	37.51–39.38	Distinct feature
IPN-3	36.57–37.98	Distinct feature
IPN-4	33.88–35.66	Distinct feature

© 2014 by the authors; licensee MDPI, Basel, Switzerland. This article is an open access article distributed under the terms and conditions of the Creative Commons Attribution license (http://creativecommons.org/licenses/by/3.0/).
